# Chemical diversity from the Tibetan Plateau fungi *Penicillium kongii* and *P. brasilianum*

**DOI:** 10.1080/21501203.2017.1331937

**Published:** 2017-06-12

**Authors:** Zhi-Guo Liu, Li Bao, Hong-Wei Liu, Jin-Wei Ren, Wen-Zhao Wang, Long Wang, Wei Li, Wen-Bing Yin

**Affiliations:** a State Key Laboratory of Mycology, Institute of Microbiology, Chinese Academy of Sciences, Beijing, China; b State Key Laboratory of Bioactive Substance and Function of Natural Medicines, Institute of Materia Medica, Chinese Academy of Medical Sciences and Peking Union Medical College, Beijing, China; c Savaid Medical School, University of Chinese Academy of Sciences, Beijing, China

**Keywords:** Antitumour cell, moulds, new compounds, structural elucidation, Tibet

## Abstract

Two new secondary metabolites, kongiilines A and B (**1, 7**), and two asperphenamate derivatives, asperphenamates B and C (**5**–**6**), together with 16 known compounds (**2**–**4, 8**–**20**), were isolated from Tibetan Plateau fungi *Penicillium kongii* and *Penicillium brasilianum*. This is the first report on asperphenamates B and C as naturally occurring compounds, and that aspterric acid is isolated from *P. brasilianum* for the first time. Their structures were elucidated by different spectroscopic techniques including high-resolution electrospray ionisation mass spectrum, 1D nuclear magnetic resonance (NMR), and 2D NMR as well as electronic circular dichroism. Compounds **4, 5,** and **10** exhibited cytotoxicity activities against human colon carcinoma HCT116 cell line with IC_50_ values of 88.16, 77.68, and 36.92 μM, respectively. Fungi from Tibetan Plateau represent important and rich resources for the investigation of new chemicals.

## Introduction

The genus *Penicillium* is a major contributor to the production of bioactive molecules. A number of compounds from this genus have been characterised and used for drugs and mycotoxins. For example, the antibiotic penicillin is produced by *Penicillium rubens* (Houbraken et al. 2011), the immunosuppressive drug mycophenolic acid is produced by *Penicillium brevicompactum* (Regueira et al. 2011). There were many new compounds that have been found constantly in the following years with impressive anticancer and antifungal activities (Bladt et al. 2013; Kozlovskii et al. 2013; Tang et al. 2015; Koul et al. 2016). And many mycotoxins causing human and animal diseases were produced in some species of *Penicillium*, such as citreoviridin, citrinin (CIT), ochratoxin A (OTA), patulin (PAT), penitrem A, and penicillic acid (PA) (Lee & Ryu 2015; Oh et al. 2017). Because of the importance of this genus, chemical assessments on more and more *Penicillium* species have been investigated from different environmental sources. The endophytic fungi (Arunpanichlert et al. 2010; Zhu et al. 2015) and soil fungi (Tansakul et al. 2014; Daengrot et al. 2015) were the most studied fungi in this genus and a number of natural products have been reported to exhibit diverse activities including anti-inflammatory, cytotoxic (Xue et al. 2014), or anti-hepatitis C virus (Kozlovskii et al. 2013; Nishikori et al. 2016). Other strains from extreme conditions also raised the great interest to mine novel structures, such as halotolerant or extremophilic strains (Lu et al. 2008; Stierle et al. 2012)，deep sea-derived strains (Li et al. 2013), and marine animal-derived strains (Qi et al. 2013). The resulted compounds cover different biological activities including cytotoxic activity against cancer cell lines (Li et al. 2014; Zhang et al. 2016), potent inhibitory activity against bacteria (Zheng et al. 2016), antifungal activity (Daengrot et al. 2015), inhibiting LPS-induced inflammation (Shin et al. 2016), and inhibitory activity against protease (Sun et al. 2014). In comparison to the above environment-derived *Penicillium* strains, research on the *Penicillium* strains from Tibetan Plateau is limited.

Tibetan Plateau, as the highest plateau in the world, is exposed to strong ultraviolet radiation, and has low-temperature and low-oxygen environments. In the past few years, a series of bioactive molecules or new skeletons has been isolated from Tibetan Plateau origin fungi, such as phaeolschidins, with antioxidant activity, from *Phaeolus schweinitzii* (Abbas et al. 2013; Han et al. 2013), new skeletons, sterhirsutins, with cytotoxic and immunosuppressant activities from *Stereum hirsutum* (Qi et al. 2014, 2015), anthraquinone derivatives, with antitumour activities, from an *Alternaria* species (Chen et al. 2014), sarcoviolins, with antioxidative and α‑glucosidase inhibitory activities, from *Sarcodon leucopus* (Ma et al. 2014a), Gloeophyllins A–J, as cytotoxic ergosteroids, from *Gloeophyllum abietinum* (Han et al. 2015). Therefore, fungi from Tibetan Plateau represent an important fungal resource for the discovery of novel chemical molecules.

In order to probe the chemical diversity from Tibetan *Penicillium*, 24 *Penicillium* strains from Tibetan Plateau were screened and evaluated for secondary metabolite production (data not shown). Based on the high-performance liquid chromatography (HPLC)-UV and/or liquid chromatography–mass spectrometry (LC-MS) analysis, two of them, *P. kongii* and *P. brasilianum* with abundant chemical profiles, were selected as targeted strains for further studies. Herein, we described the compound isolation, structural elucidation, and bioactivity evaluations for new compounds.

## Materials and methods

### Fungal materials

Plant leaf samples were collected from growing trees and kept in sterilised plastic bags. Isolation of phylloplane fungi was according to Nakase and Takashima (1993). *Penicillium* strain XZ135R was identified as *P. kongii* (isolated from leaves of *Cotoneaster* sp., Gongbujiangda, Linzhi, Tibet, China), and XZ94 was identified as *P. brasilianum* (isolated from leaves of an unidentified plant, Cuona, Shannan, Tibet, China) according to beta-tubulin gene (*BenA*, GenBank No. MF036174) and calmodulin gene (*CaM*, GenBank No. MF039288). The method of DNA extraction, the primers for amplifying the aforesaid two genes (*BenA* and *CaM*), and the polymerase chain reactions followed the methods of Wang and Wang (2013). The phylogenetic trees were drawn using maximum likelihood method and subjected to 1000 bootstrap replications (Frisvad & Samson, 2004) and supplied as Supporting Information Figures S1 and S2. The strains were stocked on slants of potato dextrose agar (PDA) at 4°C and deposited in China General Microbiological Culture Collection of Institute of Microbiology, Chinese Academy of Sciences, Beijing, China as AS3.15332 and AS3.15722, respectively, and they were also maintained at the authors’ laboratory and will be supplied upon request for educational or scientific purpose. The two strains were activated and cultured on plates of PDA at 25°C for 7 d. Then, cultures were transferred to the modified YES solid medium (2% yeast extracts (BD Bacto^TM^), 15% sucrose, and 1.5% agar) with 20.0 mL on each plate at 25°C for 7 d (Frisvad 1981). For the large-scale fermentations, 12 L YES solid cultures of *P. kongii* and 3 L YES solid cultures of *P. brasilianum* were performed, respectively. They were growing at 25°C for 7 d before harvest.

### Analysis methods

Analytical HPLC was conducted with a Waters HPLC system (Waters e2695, Waters 2998, Photodiode Array Detector) using an ODS column (C18, 250 mm × 4.6 mm, YMC Pak, 5 μM) with a flow rate of 1 mL/min. Fresh extracts were dissolved in methanol before separated on a linear gradient of MeOH:H_2_O (0.1% formic acid) at a flow rate of 1 mL/min. Fresh extracts from screened strains were detected for 30 min using a linear gradient of 20–100% (0–20 min), 100% MeOH (20–25 min), 20% MeOH (25–30 min). LC-MS analysis method was used as keeping consistent with analytical HPLC.

### General experimental procedures

The optical rotations were measured on a Perkin-Elmer 241 polarimeter (Waltham, USA) and UV spectra were determined on a Thermo Genesys-10S UV–Vis spectrophotometer (Madison, USA). Electronic circular dichroism (ECD) spectra were recorded on a JASCO J-815 spectropolarimeter (Tokyo, Japan). NMR spectra were recorded on a Bruker Avance-500 spectrometer using tetramethyl silicane (TMS) as internal standard, and chemical shifts were recorded as δ values. High-resolution electrospray ionisation mass spectrum (HR-ESI-MS) and LC-MS were utilised on an Agilent Accurate-Mass-QTOF LC-MS 6520 instrument. Sephadex LH-20 was purchased from GE Healthcare. TLC was carried out on Silica gel HSGF254 and the spots were visualised by spraying with 10% H_2_SO_4_ and heating. Silica gel (Qingdao Haiyang Chemical Co., Ltd., Qingdao, China) and ODS (Lobar, 40–63 mm, Merck, Darmstadt, Germany) were used for column chromatography (CC). RP-HPLC separations were conducted using a Shimadzu LC-6AD liquid chromatograph with a YMC-PACK ODS-A column (250 mm × 10 mm, 5 µm) and a Shimadzu SPD-20A VP UV–Vis detector with a flow rate of 3 mL/min. Solvents used for extraction and chromatographic separation were of analytical grades.

### Extraction and isolation

The fermented YES cultures were extracted thoroughly with ethyl acetate (2 × 12 L and 2 × 3 L, respectively) under ultrasonic with 100 Hz for 1 h, and then, organic phases were evaporated to dryness under reduced pressure to afford two crude residues (9.8 g and 3.0 g, respectively). The 9.8-g residues of *P. kongii* were subjected to a silica gel CC using a gradient elution with CH_2_Cl_2_–MeOH (100:0–0:100, v/v) to give six fractions (fractions A–F). Fr. A (1.87 g) was subjected to Sephadex LH-20 CC eluting with MeOH to afford two subfractions (Fr. A-1–Fr. A-2). Fr. A-1 was repeatedly recrystallised in CHCl_3_–MeOH (1:1) to give compound **4** (180.0 mg) and **10** (220.0 mg). Compound **3** (30 mg, tR 23.5 min) was purified from Fr. A-2 by semi-preparative HPLC (MeOH–H_2_O, 75:25). Fr. B (0.89 g) was divided into three subfractions Fr. B-1–Fr. B-3 by Sephadex LH-20 column (MeOH). Fr. B-1 was further purified by semi-preparative HPLC (MeOH–H_2_O, 70:30) to afford **8** (5.9 mg, tR 11.9 min), **2** (5.6 mg, tR 14.3 min), **1** (4.3 mg, tR 19.8 min), **5** (10.0 mg, tR 23.1 min), and **6** (1.8 mg, tR 29.8 min). Compounds **9** (7.4 mg, tR 18.5 min) and **11** (4.7 mg, tR 22.4 min) were isolated from Fr. B-3 by semi-preparative HPLC eluting with MeOH–H_2_O (45:55). Fr. C (1.03 g) was passed through a Sephadex LH-20 column (MeOH) and further purified by semi-preparative HPLC (MeOH–H_2_O, 60:40) to afford **7** (1.7 mg, tR 15.9 min).

The 3.0-g extracts of *P. brasilianum* were subjected to CC on reversed-phase ODS using a gradient system of MeOH–H_2_O (from 40% to 100%) to afford seven fractions (Fr. G–Fr. M). Fr. H (0.5 g) was divided into three subfractions Fr. H-1–Fr. H-3 by Sephadex LH-20 column eluting with MeOH. Compounds **18** (12.4 mg, tR 14.6 min), **12** (2.7 mg, tR 17.5 min), and **15** (4.9 mg, tR 22.9 min) were purified from Fr. H-1 by semi-preparative HPLC (MeOH–H_2_O, 60:40). Fr. J (0.4 g) was passed through a Sephadex LH-20 column (MeOH) and further purified by semi-preparative HPLC (MeOH–H_2_O, 65:35) to afford **16** (0.9 mg, tR 21.4 min) and **17** (3.0 mg, tR 24.5 min). Fr. K (0.3 g) was chromatographed on a Sephadex LH-20 column eluting with MeOH to give **19** (2.3 mg) and **20** (13.7 mg). Fr. M (0.8 g) was subjected to a silica gel column eluting with petroleum ether–acetone, followed by recrystallisation in CHCl_3_ to give **13** (280 mg) and **14** (60 mg). Kongiiline A (**1**), white needles.[a]^25^_D__–_65 (*c* 0.1, MeOH). UV (MeOH) *λ*_max_ (log *ε*) 238 (2.80) nm; 273 (0.72) nm.^1^H NMR (500 MHz, methanol-*d*_4_) and ^13^C NMR (125 MHz, methanol-*d*_4_) see Table 1. HR-ESI-MS *m/z* 373.2013 [M + H]^+^ (calcd for C_22_H_29_O_5_, 373.2010).

**Table 1.** ^1^H NMR and ^13^C NMR data for compounds **1** and **7.**

	1a			7b	
Position	*δ*_C_	*δ*_H_ (*J* Hz)	Position	*δ*_C_	*δ*_H_ (*J* Hz)
1a	43.8	1.12, m	2	161.6	
1b		1.18, m	3	87.5	5.65, s
2a	18.7	1.53, m	4	169.1	
2b		1.76, m	5	149.2	
3a	38.3	1.20, m	6	107.7	7.26, s
3b		1.69, m	7	131.4	
4	39.2		8	143.7	
5	45.9	1.80, m	9	132.1	
6	69.3	5.67, m	10	105.9	
7a	28.8	2.12, m	11	65.4	4.67, d (6.5)
7b		2.47, m	12	119.8	5.47, d (6.5)
8	37.0	2.87, t (8.0)	13	137.3	
9	50.7	2.40, m	14	18.1	1.74, s
10	35.7		15	25.5	1.76, s
11a	69.4	4.28,dd (5.5,10)	16	61.2	4.80, d (4.0)
11b		4.47, d (9.5)	17	56.7	3.94, s
12	181.2				
13	20.3	0.88, s			
14a	71.2	3.03, d (6.5)			
14b		3.56, d (6.0)			
15	19.1	1.43, s			
1′	168.1				
2′	132.0				
3′	130.6	7.95,dd (1.5,7.0)			
4′	129.6	7.48, d (8.0)			
5′	134.1	7.60, t (7.5)			
6′	129.6	7.47, d (7.5)			
7′	130.6	7.97, dd (1.5,7.0)			

^a^Recorded in MeOD. ^b^recorded in DMSO-*d*_6_.

Asperphenamate B (**5**), white powder.[a]^25^_D__–_124 (*c* 0.1, MeOH). UV (MeOH) *λ*_max_ (log *ε*) 237 (2.38) nm; 272 (0.26) nm. ECD (MeOH) λ_max_ (Δε) 234 (−9.28) nm. ^1^H NMR (500 MHz, DMSO-*d*_6_) and ^13^C NMR (125 MHz, DMSO-*d*_6_) see Table 2. HR-ESI-MS *m/z* 523.2232 [M + H]^+^ (calcd for C_32_H_31_N_2_O_5_, 523.2227).

**Table 2.** ^1^H NMR and ^13^C NMR data for compounds **5** and **6.**

	5a		6b	
Position	*δ*_C_	*δ*_H_ (*J* Hz)	*δ*_C_	*δ*_H_ (*J* Hz)
1	172.2		173.3	
2	54.8	4.87, dd (6.5,11.5)	56.3	4.76, dd (6.0,9.0)
3a	36.9	3.12, dd (6.5,14.0)	37.9	3.13, dd (9.0,14.0)
3b		3.19, dd (6.5,14.0)		3.27, dd (6.0,9.0)
4	127.4		138.6	
5	130.5	7.04, d (8.5)	130.5	7.26, m
6	116.0	6.76, d (8.5)	129.5	7.24, m
7	155.5		127.9	7.22, m
8	116.0	6.76, d (8.5)	129.5	7.24, m
9	130.5	7.04, d (8.5)	130.5	7.26, m
10	167.7		170.3	
11	134.2		125.7	
12	127.3	7.68, d (7.5)	130.2	7.60, d (8.5)
13	128.8	7.30, t (7.5)	116.1	6.76, d (8.5)
14	131.6	7.42, t (7.5)	162.3	
15	128.8	7.30, t (7.5)	116.1	6.76, d (8.5)
16	127.3	7.68, d (7.5)	130.2	7.60, d (8.5)
1′a	65.5	4.03, dd (3.5,11.5)	66.7	4.11, dd (6.0,11.0)
1′b		4.50, dd (4.5,11.0)		4.41, dd (4.5,11.0)
2′	50.5	4.60, m	51.9	4.56, m
3′a	37.4	2.90, dd (7.0,14.0)	37.4	2.88, dd (8.5,14.0)
3′b		3.00, dd (6.5,14.0)		2.93, dd (6.0,14.0)
4′	137.2		139.1	
5′	129.4	7.22, d (8.0)	130.3	7.25, m
6′	128.9	7.30, t (7.5)	129.4	7.23, m
7′	127.0	7.30, t (7.5)	127.6	7.20, m
8′	128.9	7.24, t (7.5)	129.4	7.23, m
9′	129.4	7.22, d (8.0)	130.3	7.25, m
10′	167.6		170.1	
11′	133.4		135.7	
12′	127.2	7.65, d (7.5)	128.4	7.67, d (7.5)
13′	128.6	7.38, t (7.5)	127.2	7.37, d (7.5)
14′	132.2	7.50, t (7.5)	132.6	7.49, t (7.5)
15′	128.6	7.38, t (7.5)	127.2	7.37, d (7.5)
16′	127.2	7.65, d (7.5)	128.4	7.67, d (7.5)

^a^Recorded in DMSO-*d*_6._^b^recorded in MeOD.

Asperphenamate C (**6**), white powder.[a]^25^_D__–_89 (*c* 0.1, MeOH). UV (MeOH) *λ*_max_ (log *ε*) 242 (1.20) nm. ECD (MeOH) λ_max_ (Δε) 234 (−3.89) nm. ^1^H NMR (500 MHz, methanol-*d*_4_) and ^13^C NMR (125 MHz, methanol-*d*_4_) see Table 2. HR-ESI-MS *m/z* 523.2231 [M + H]^+^ (calcd for C_32_H_31_N_2_O_5_, 523.2227).

Kongiiline B (**7**), white needles. UV (MeOH) *λ*_max_ (log *ε*) 248 (0.32) nm; 306 (0.54) nm. ^1^H NMR (500 MHz, DMSO-*d*_6_) and ^13^C NMR (125 MHz, DMSO-*d*_6_) see Table 1. HR-ESI-MS *m/z* 307.1172 [M + H]^+^ (calcd for C_16_H_19_O_6_, 307.1176).

### Cytotoxicity assays

Cytotoxic activity against HCT116 human colon carcinoma cells was assayed according to the 3-[4,5-dimethylthiazol-2-yl]-2,5 diphenyl tetrazolium bromide (MTT) assay method (Ma et al. 2014b). Cells were incubated with tested compounds (DMSO as solvent) at 37°C in a humidiﬁed atmosphere of 5% CO_2_ 95% air for 72 h. Each well was added 50 μL of MTT/medium solution (0.5 mg/mL), and cells were incubated for another 4 h. After removing the MTT/medium, 100 μL of DMSO was added to each well. The plate was shaken to dissolve the precipitates, and activity was measured at 540 nm using a microplate reader. The inhibition rates were calculated and plotted versus test concentrations to aﬀord the IC_50_ (± SD) for three independent experiments, each was carried out in triplicate. Taxol was used as the reference substance that showed cytotoxicity against HCT116 human colon carcinoma with IC_50_ value of 0.98 ± 0.12 μM.

## Results and discussion

**Figure 1.**
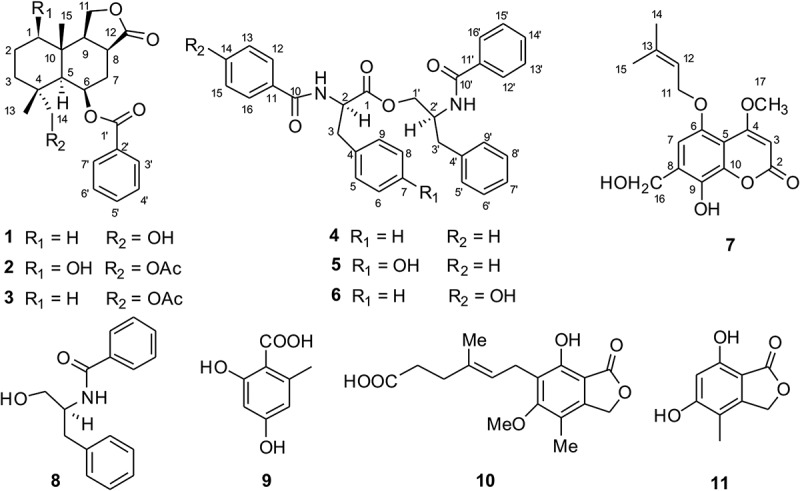
The chemical structures of compounds 1–11 from *P. kongii.*

**Figure 2.**
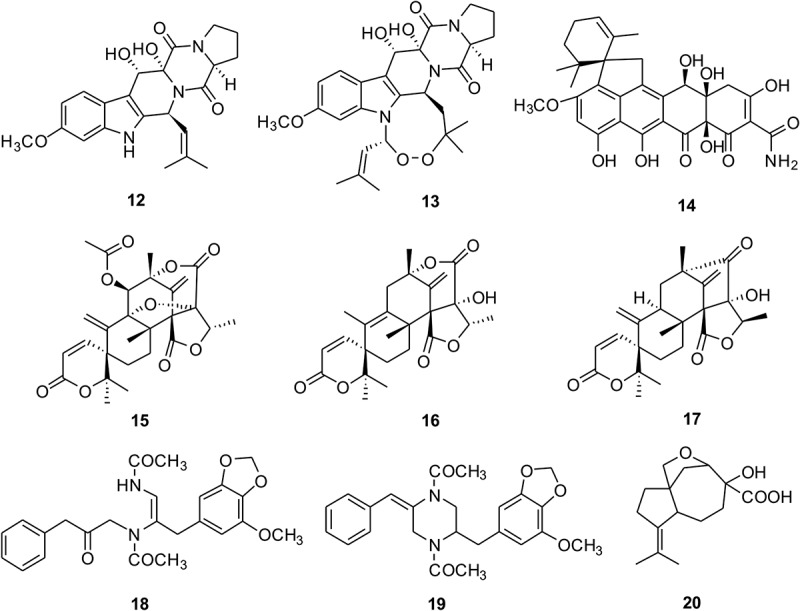
The chemical structures of compounds 12–20 from *P. brasilianum.*

To characterise the compounds from *P. kongii* and *P. brasilianum*, the fermentations on YES media were carried out. The organic extracts were fractionated by ODS and followed from Sephadex LH-20 CC (see Methods). The subfractions containing the targeted metabolites were then selected for further purification. After the semi-preparative reversed-phase HPLC separation step, we isolated 20 compounds (**1**–**20**) including 2 new compounds which were named kongiilines A and B (**1, 7**) and 2 new natural compounds which were named asperphenamates B and C (**5, 6**) (Figures 1 and 2). The two compounds of asperphenamates B and C (**5, 6**), as asperphenamate derivatives, were synthesised in 2012 (Yuan et al. 2012), but they were isolated from fungi as the natural compounds for the first time.

**Figure 3.**
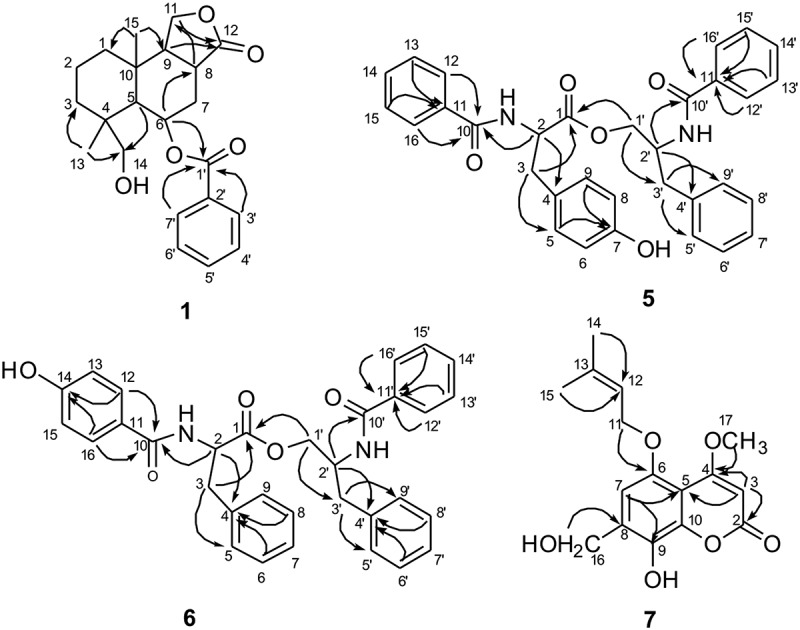
The key HMBC correlations of compounds 1, 5–7.

Kongiiline A (**1**) was isolated as white needles. The molecular formula C_22_H_29_O_5_ of **1** was determined by HR-ESI-MS at *m/z* 373.2013 [M + H]^+^ (calcd 373.2010). ^1^H NMR spectrum (Table 1) indicated the presence of two tertiary methyls (*δ* 0.88, 1.43), two oxymethylenes (*δ* 3.03, d, *J *= 6.5 Hz; 3.56, d, *J *= 6.0 Hz; 4.28, dd, *J *= 10.0, 5.5 Hz; 4.47, d, *J *= 9.5 Hz), one oxymethine (*δ* 5.67, m), and five aromatic protons, respectively. ^13^C NMR and heteronuclear singular quantum correlation (HSQC) spectra showed a total of 22 carbon signals that could be classified into 2 carbonyl, 6 olefinic carbons, 3 sp^3^ quaternary carbon, 5 sp^3^ methines (including 2 oxymethines), 4 sp^3^ methylenes, and 2 methyls (Table 1). Analysing the ^1^H and ^13^C NMR spectra indicated that **1** possessed the drimane-type sesquiterpenoid skeleton, which showed signals similar to those for 1-deoxypebrolide (McCorkindale et al. 1978). The sole difference was that one acetyl group is replaced by a hydroxyl group at C-14 in **1**. This was corroborated by analysis of the heteronuclear multiple bond correlation (HMBC) spectrum, in which long-range correlations of H-5 and H_3_-13 with C-14 (*δ* 71.2), and of H-14 to C-4 (*δ* 39.2), C-5 (*δ* 45.3) and C-13 (*δ* 20.3), indicated that the hydroxyl group located at c-14(*δ* -71.2) (Figure 3). The relative configuration of **1** was further confirmed by analysis of the coupling constants and nuclear overhauser effect spectroscopy (NOESY) spectrum, in which the correlations of H-14/H-5 and H-6, H-5/H-9, H-6/H-8, and H_3_-13/H_3_-15 indicated an *α*-orientation of H-5, H-6, H-8, H-9, H_2_-14 and a *β*-orientation of H_3_-13 and H_3_-15, respectively. The structure of **1** was finally named as1-deoxy-14-desacetylpebrolide.

Asperphenamate B (**5**) was isolated as white powder, and the molecular formula was determined to be C_32_H_30_N_2_O_5_ based on the HR-ESI-MS at m/z 523.2232 [M + H]^+^ (calcd for C_32_H_31_N_2_O_5_, 523.2227). The NMR data (Table 2) suggested that the structure of compound **5** was likely an amino-acid ester, which was similar to that of asperphenamate (Fukuda et al. 2011). The difference between the two compounds was that an additional hydroxyl group was located at **5**. From the HMBC spectrum analysis, the correlations from H-2 to C-1 (*δ* 172.2), C-3 (*δ* 36.9), and C-10 (*δ* 167.7) and from H-3 to C-1 (*δ* 172.2), C-5 (*δ* 130.5), and C-9 (*δ* 130.5) and from H-5/H-9 to C-3 (*δ* 36.9), C-4 (*δ* 127.4), and C-7 (*δ* 155.5) and from H-12/H-16 to C-10 (*δ* 167.7) indicated the partial structure with a N-benzoyl-tyrosine group (Figure 3). The partial structure of N-benzoyl-phenylalaninol was defined by the HMBC correlations from H-2′ to C-1′ (*δ* 65.5) and C-10′ (*δ* 167.6), and from H-3′ to C-1′ (δ 65.5), C-5′ (δ 129.4), and C-9′ (δ 129.4) and from H-1′ to C-2′ (δ 50.5) and C-3′ (δ 37.4), and from H-12′/H-16′ to C-10′ (δ 167.6) (Figure 3). In addition, its absolute configuration was established from the negative Cotton effect at 234 nm in the ECD spectrum, which was consistent with a known compound asperphenamate (McCorkindale et al. 1978). Furthermore, the specific rotation of **5** was similar to that of asperphenamate. Therefore, the three-dimensional (3D) configurations of 2 and 2′ position are same between compound **5** and asperphenamate, and the structure of **5** was defined as N-benzoyl-tyrosine-2-benzoylamino-3-phenyl propyl ester.

Asperphenamate C (**6**) has the same molecular formula C_20_H_30_N_2_O_5_ with *m/z* 523.2231 [M + H]^+^ (calcd for C_32_H_31_N_2_O_5_, 523.2227) as **5** based on the HR-ESI-MS analysis. Comparison of the 1D and 2D NMR data between **6** and **5** indicated that the gross structure of compound **6** is the same as that of **5** with the difference of the location of one of the hydroxyl groups (Table 2). The hydroxyl group was determined at C-14 from the observation of the HMBC correlations from H-12/H-16 to C-10 (*δ* 170.3), C-13/C-15 (*δ* 116.1), and C-14 (*δ* 162.3). The absolute configuration of **6** was determined by the ECD spectrum, which showed the similar Cotton effect with asperphenamate. Furthermore, the specific rotation of **5** was similar to that of asperphenamate, which was consistent with asperphenamate (McCorkindale et al. 1978). Thus, the 3D configurations of 2 and 2′ position are same between compound **6** and asperphenamate, and compound **6** was defined as N-p-phydroxybenzoyl-phenylalanine-2-benzoylamino-3-phenyl propyl ester. To the best of our knowledge, compound **6** was isolated from nature for the first time.

Kongiiline B (**7**) was obtained as white needles. Its molecular formula was determined to be C_16_H_18_O_6_ with *m/z* 307.1172 [M + H]^+^ (calcd for C_16_H_19_O_6_, 307.1176) based on HR-ESI-MS data. Analyses of the ^1^H, ^13^C NMR data (Table 1) and HSQC spectra of **7** indicated that it belongs to isopentenylcoumarin (Fukuda et al. 2011), which was bearing substituent groups of methylol, methoxy, and hydroxyl. The prenyloxy group was attached to C-5 by the analysis of HMBC correlations (Figure 3) from H_2_-11 to C-5 (δ 149.2). The relative configuration of **7** was further confirmed by analysis of the coupling constants and NOESY spectrum. The long-range correlations from H_2_-16 to C-6 (*δ* 107.7), C-7 (*δ* 131.4) and C-8 (*δ* 143.7), and from H_3_-17 to C-4 (*δ* 169.1) indicated that methylol group was at C-7, hydroxyl group at C-9, and methoxy group at C-4, respectively. Thus, the structure of compound **7** was established as 3-methoxy-7-methylol-8-hydroxy-5- isopentenylcoumarin.

The other known compounds isolated from *P. kongii* including pebrolide (Mccorkindale et al. 1981) (**2**), 1-deoxypebrolide (Mccorkindale et al. 1981) (**3**), asperphenamate (McCorkindale et al. 1978) (**4**), N-benzoyl-phenylalaninol (McCorkindale et al. 1978) (**8**), orsellinic acid (Fang et al. 2012) (**9**), mycophenolic acid (Makara et al. 1996) (**10**), and 5, 7-dihydroxy-4-methylphthalide (Fujimoto et al. 1999) (**11**), and the other known compounds isolated from *P. brasilianum* including 12, 13-dihydroxyfumitremorgin C (Inokoshi et al. 2013) (**12**), verruculogen (Afiyatullov et al. 2004) (**13**), viridicatumtoxin (Inokoshi et al. 2013) (**14**), dehydroaustin (Hayashi et al. 1994) (**15**), austinolide (Lo et al. 2012) (**16**), neoaustin (Hayashi et al. 1994) (**17**), brasiliamide A (Fujita et al. 2002) (**18**), brasiliamide C (Fujita & Hayashi 2004) (**19**), and aspterric acid (Shimada et al. 2002) (**20**), were determined by comparison of those reported spectroscopic data. Among these, aspterric acid is reported from *P. brasilianum* for the first time. There was obvious difference on metabolic profiles between the *P. brasilianum* strain from plant leaves of Tibetan Plateau environments and those as endophytes (Tang et al. 2015) and soil isolates (Fujita & Hayashi, 2004).

The isolated compounds were tested for their cytotoxicities against human cancer cells (Table 3). Hydroxylated asperphenamates asperphenamate B (**5**) and asperphenamate C (**6**) displayed very weak cytotoxicity activities against human cancer cell line HCT116. The IC_50_ values for **5** and **6** were detected at 88.16 and 77.68 μM, respectively (Table 3). Known compound mycophenolic acid (**10**) exhibited activities against HCT116 with IC_50_ value of 36.92 μM (Table 3). This compound has shown previously outstanding anti-proliferative immunosuppressive activity and was used as drug for organ transplant patients. The other tested compounds were inactive.

**Table 3.** Cytotoxic activities against HCT116 of **1–11.**

Compound	IC_50_ ± SD (μM)	Compound	IC_50_ ± SD (μM)
**1**	>100	**7**	>100
**2**	>100	**8**	>100
**3**	>100	**9**	>100
**4**	88.16 ± 5.52	**10**	36.92 ± 1.96
**5**	77.68 ± 2.84	**11**	>100
**6**	91.72 ± 8.31	Taxol	0.98 ± 0.12

Among these compounds, asperphenamate is composed of two amino acids via ester bond connection and has been reported with anticancer activity (Pomini et al. 2006). Subsequently, several derivatives were synthesised for improving the solubility and activity. For example, asperphenamate derivatives **1a** and **1c**, as a pair of isomers, showed activities against T47D, MDA-MB231, and HL60 cell lines, and **1c** exhibited the most potent activities against breast cancer cell lines T47D and MDA-MB231 (IC_50_ = 8.2 and 11.9 μM, respectively) (Yuan et al. 2010). Asperphenamate derivative IM23b, benzyl modified at C15 of asperphenamate, showed the greatest potency in human breast cancer MCF-7 cells (Yuan et al. 2012). This compound was also found in *P. brevicompactum*. The phylogenetic and morphological study showed that *P. kongii* belongs to *Penicillium* section *brevicompacta* and has some kinship with *P. brevicompactum* (Wang & Wang 2013). Recently, genome of *P. brasilianum* has been sequenced (Horn et al. 2015), which provides the opportunity to elucidate its biosynthetic pathway.
