# Supplementary material for: Chemical diversity from the Tibetan Plateau fungi Penicillium kongii and P. brasilianum
Source: Mycology. 2017 Jun 12;9(1):10–9. doi: 10.1080/21501203.2017.1331937 (PMC6059045; doi:10.1080/21501203.2017.1331937)
Supplement: Supp_Data_1331937.doc [file TMYC_A_1331937_SM3811.doc]

**Supporting Information Contents**

**Figure S1.** The phylogenetic tree inferred from *CaM* sequences.

**Figure S2.** The phylogenetic tree inferred from *BenA* sequences.

**Figure S3.** HPLC analyses for crude extracts from *P*. *kongii* and *P*. *brasilianum.*

**Figure S4.** ^1^H (500 MHz) NMR spectrum of compound **1** in MeOD.

**Figure S5.** ^13^C (125 MHz) NMR spectrum of compound **1** in MeOD.

**Figure S6.** HSQC spectrum of compound **1** in MeOD.

**Figure S7.** HMBC spectrum of compound **1** in MeOD.

**Figure S8.** NOESY spectrum of compound **1** in MeOD.

**Figure S9.** ^1^H (500 MHz) NMR spectrum of compound **5** in DMSO-d_6_.

**Figure S10.** ^13^C (125 MHz) NMR spectrum of compound **5** in DMSO-d_6_.

**Figure S11.** ECD spectrum of compound **5** in MeOH.

**Figure S12.** ^1^H (500 MHz) NMR spectrum of compound **6** in MeOD.

**Figure S13.** ^13^C (125 MHz) NMR spectrum of compound **6** in MeOD.

**Figure S14.** ECD spectrum of compound **6** in MeOH.

**Figure S15.** ^1^H (500 MHz) NMR spectrum of compound **7** in DMSO-d_6_.

**Figure S16.** ^13^C (125 MHz) NMR spectrum of compound **7** in DMSO-d_6_.

**Figure S17.** HSQC spectrum of compound **7** in DMSO-d_6_.

**Figure S18.** HMBC spectrum of compound **7** in DMSO-d_6_.

**Figure S19.** NOESY spectrum of compound **7** in DMSO-d_6_.

**Figure S1.** The phylogenetic tree inferred from *CaM* sequences.

**
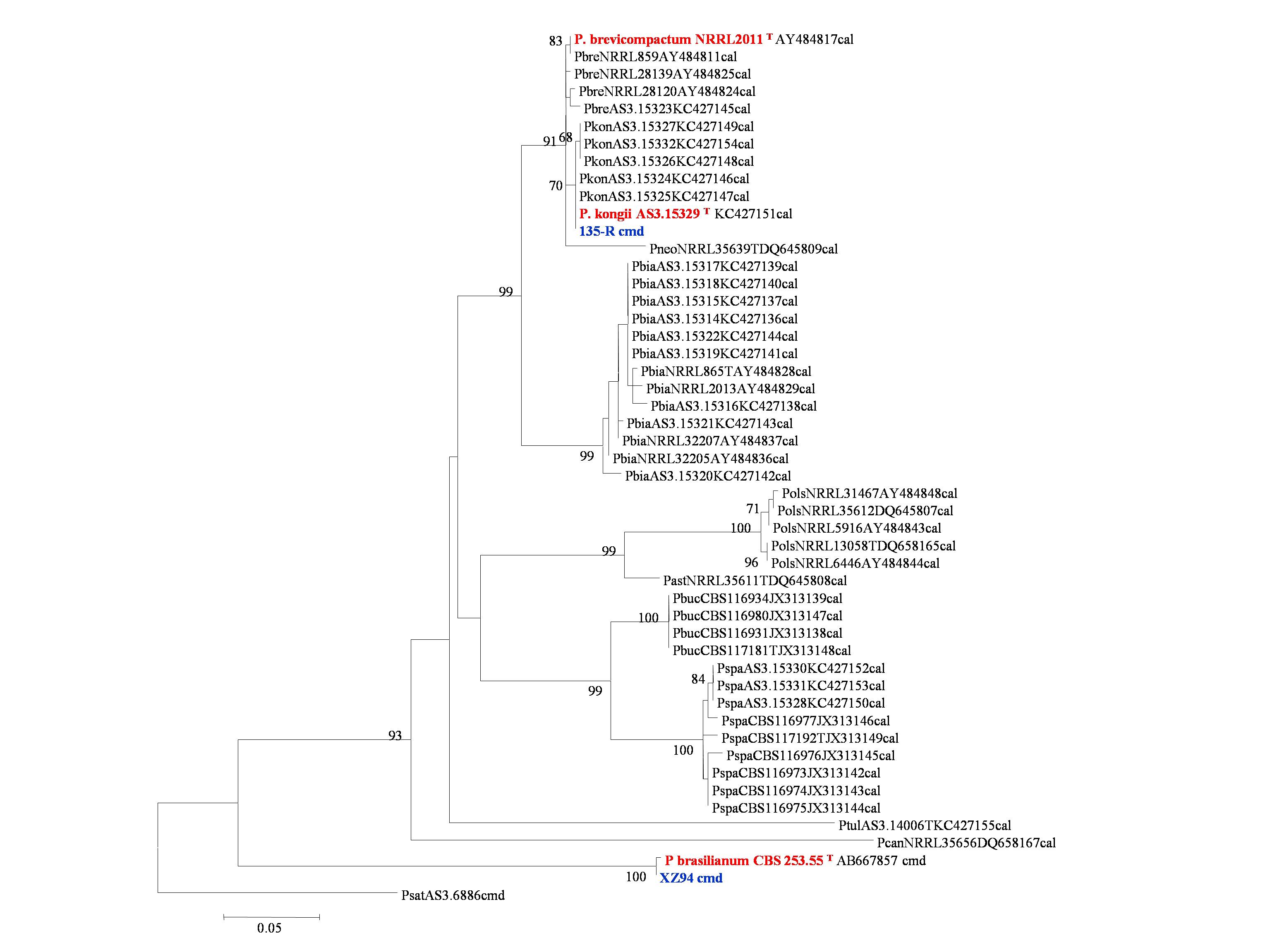
**

**Figure S2.** The phylogenetic tree inferred from *BenA* sequences.

**
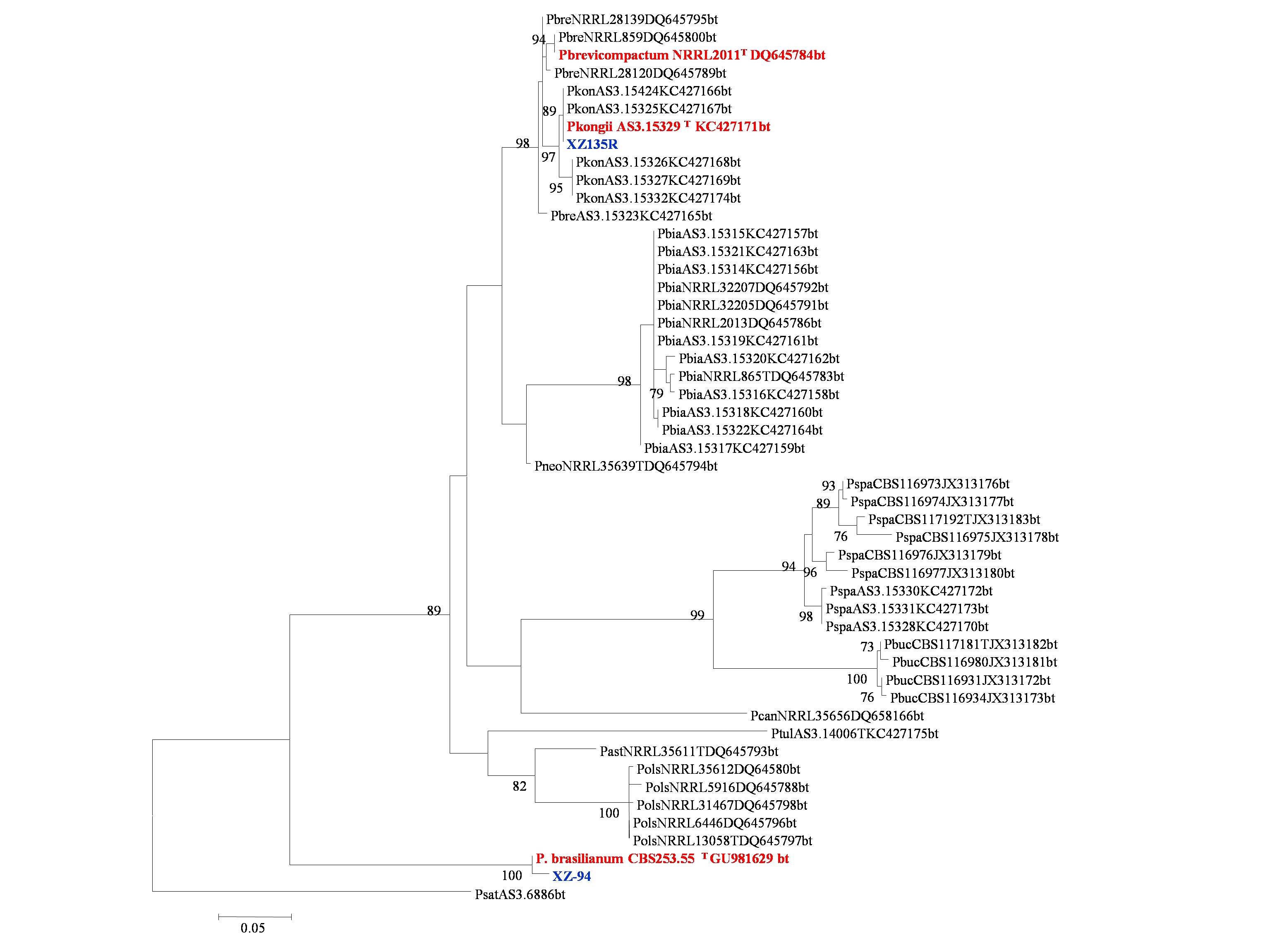
**

**Figure S3.** HPLC analyses for crude extracts from *P*. *kongii* and *P*. *brasilianum*


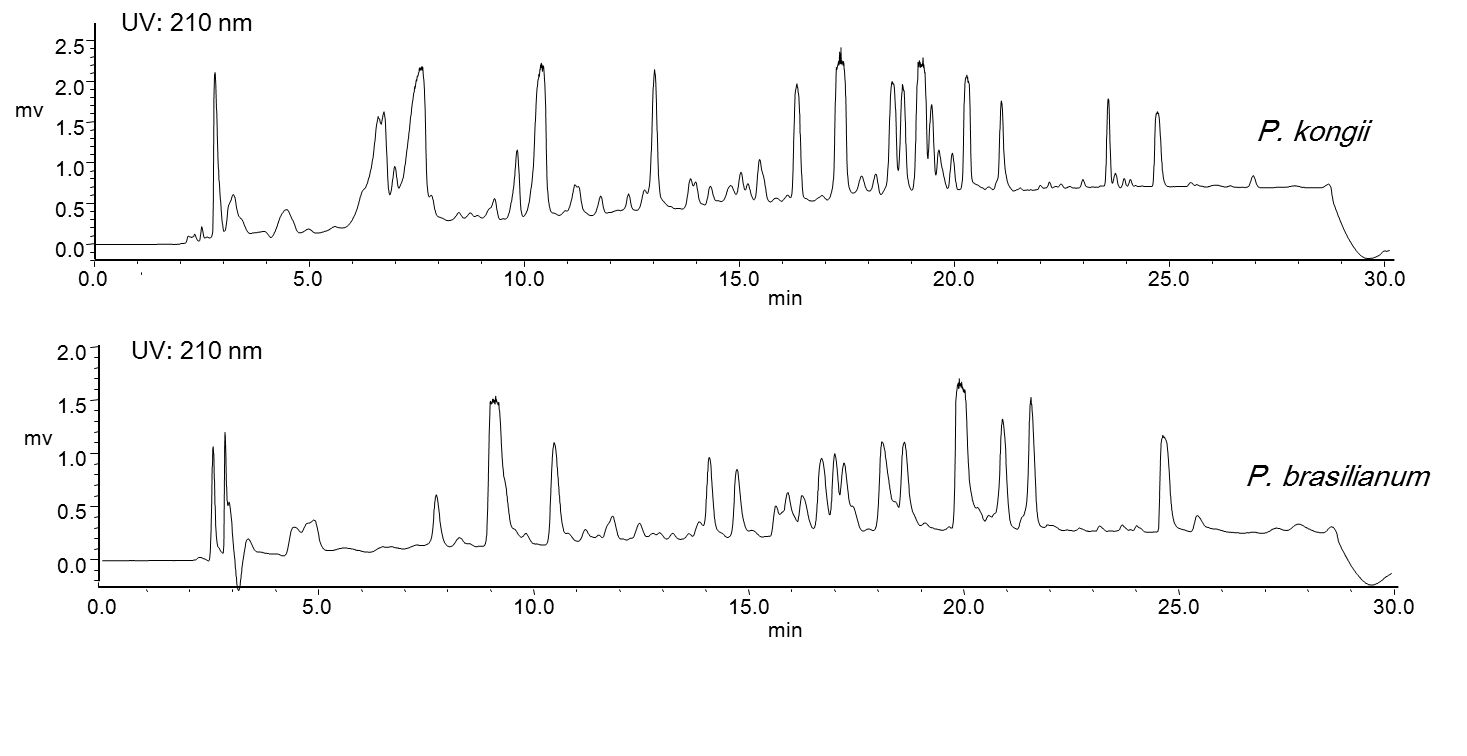


**Figure S4.** ^1^H (500 MHz) NMR spectrum of compound **1** in MeOD
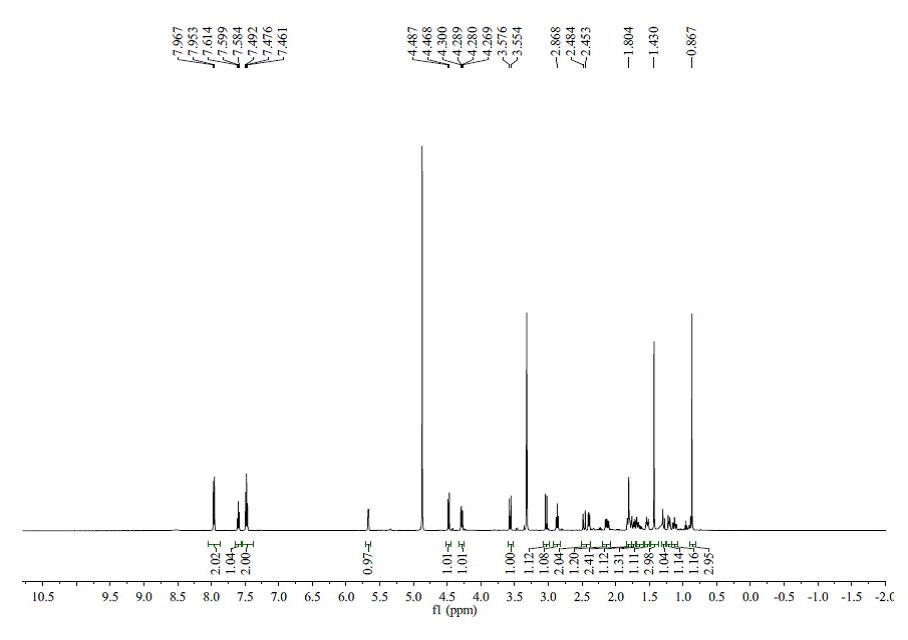


**Figure S5.** ^13^C (125 MHz) NMR spectrum of compound **1** in MeOD
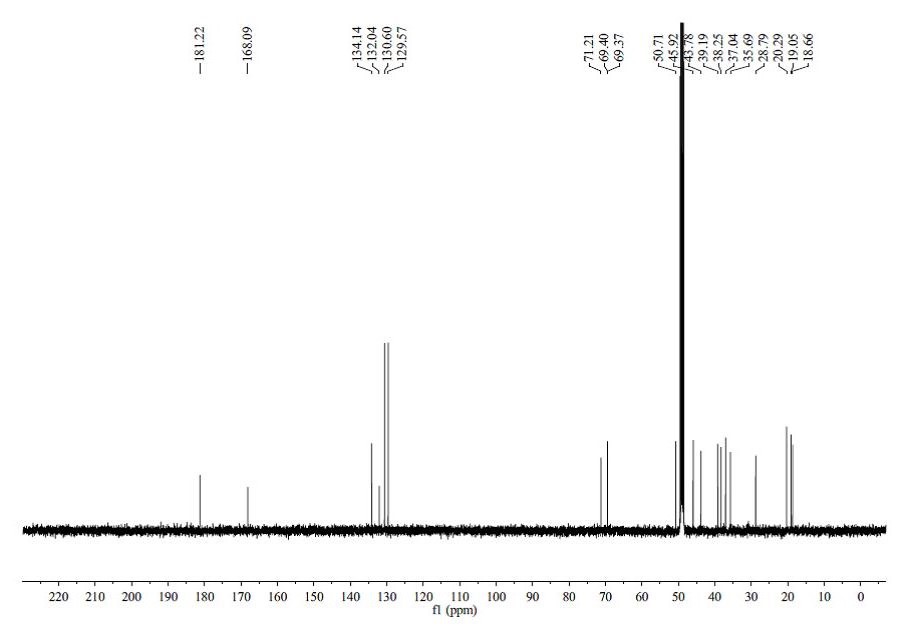


**Figure S6.** HSQC spectrum of compound **1** in MeOD

**Figure S7.** HMBC spectrum of compound **1** in MeOD

**Figure S8.** NOESY spectrum of compound **1** in MeOD

**Figure S9.** 1H (500 MHz) NMR spectrum of compound **5** in DMSO-d6


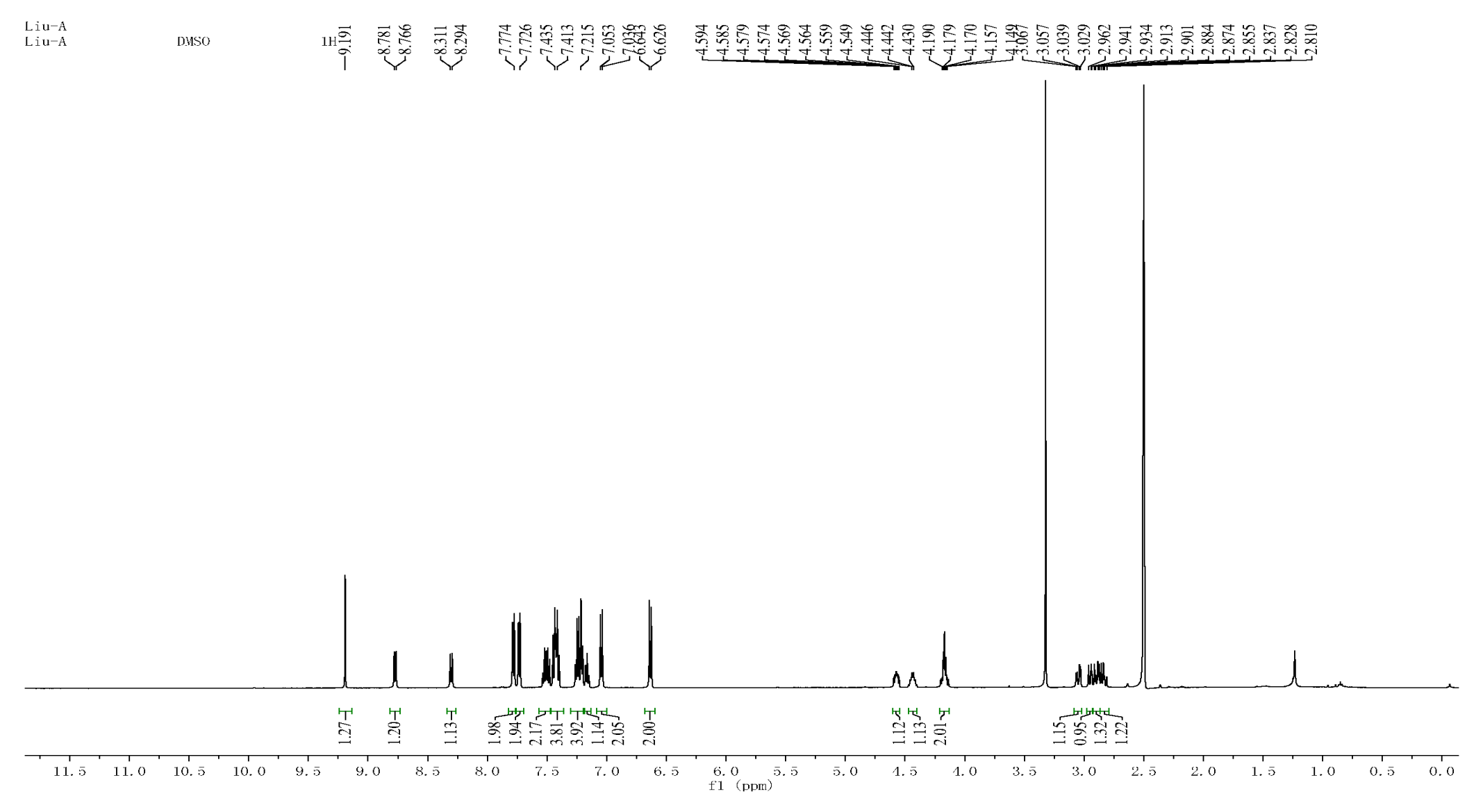


**Figure S10.** ^13^C (125 MHz) NMR spectrum of compound **5** in DMSO-d_6_


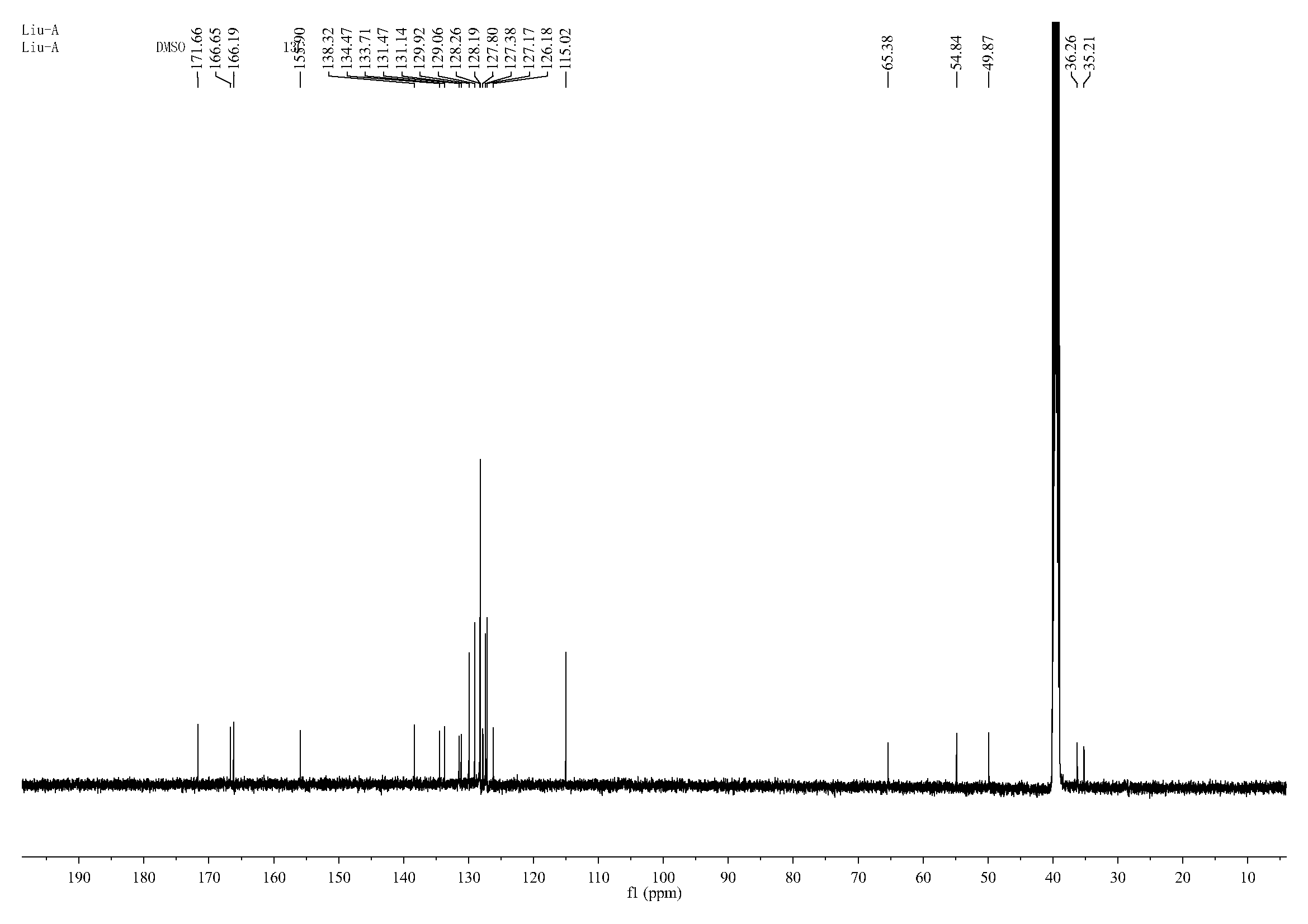


**Figure S11.** ECD spectrum of compound **5** in MeOH


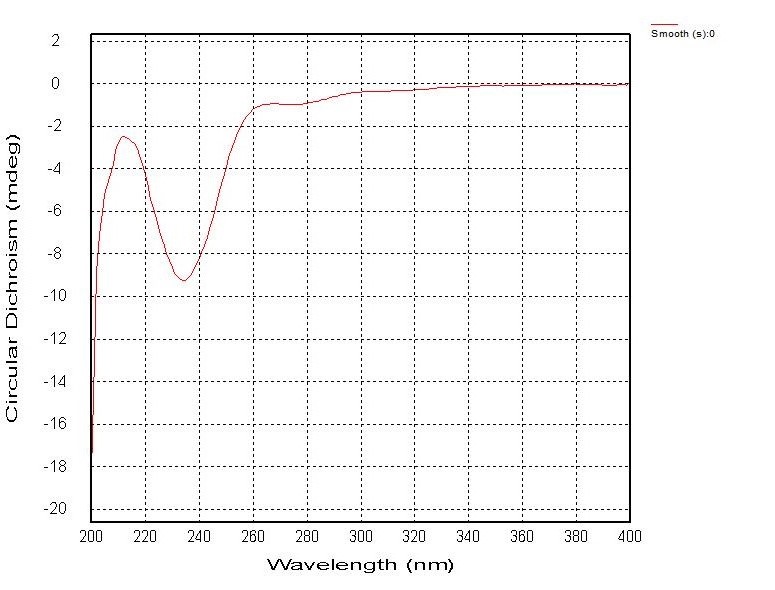


**Figure S12.** ^1^H (500 MHz) NMR spectrum of compound **6** in MeOD


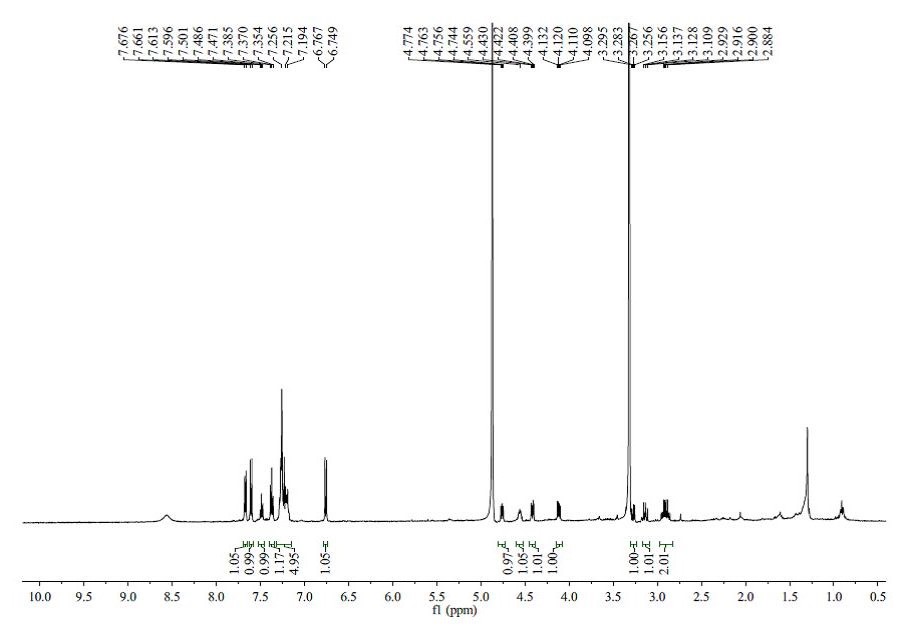


**Figure S13.** ^13^C (125 MHz) NMR spectrum of compound **6** in MeOD


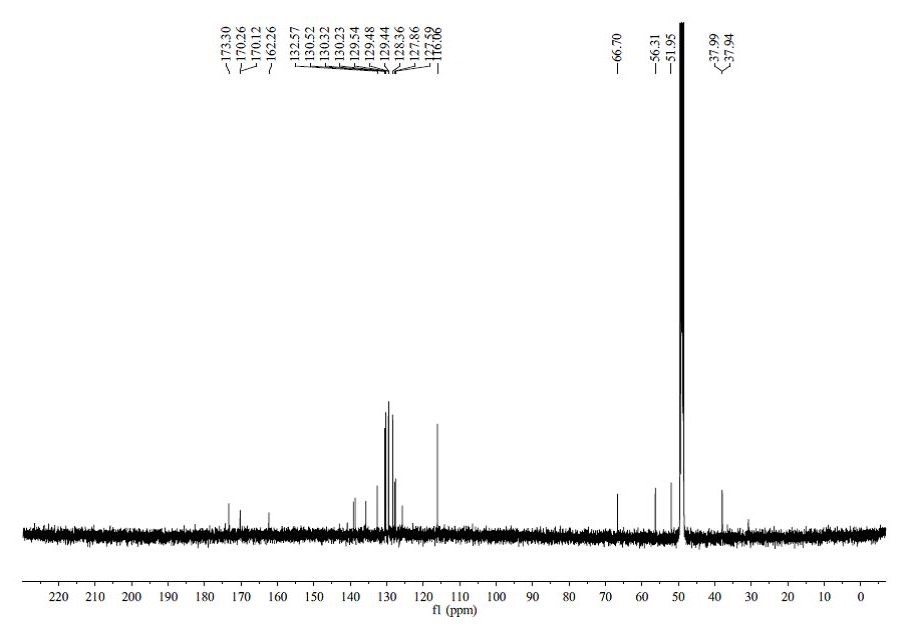


**Figure S14.** ECD spectrum of compound **6** in MeOH


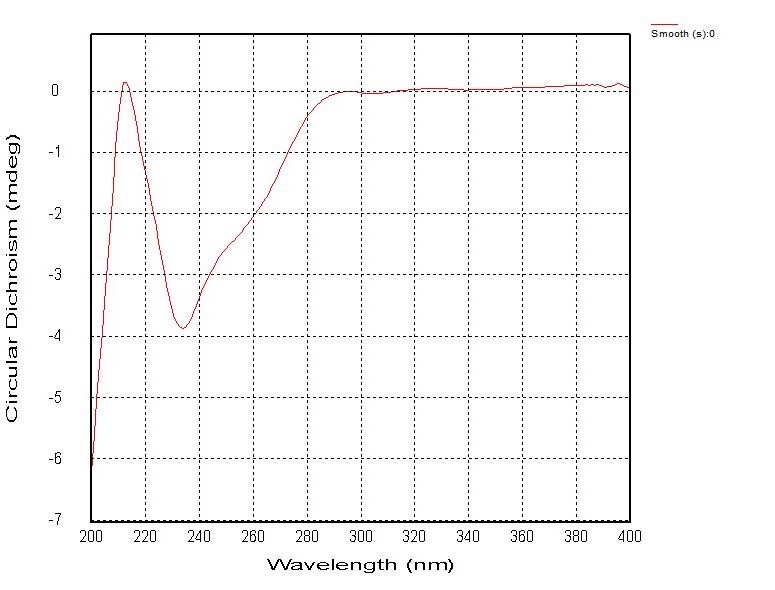


**Figure S15.** ^1^H (500 MHz) NMR spectrum of compound **7** in DMSO-d_6_


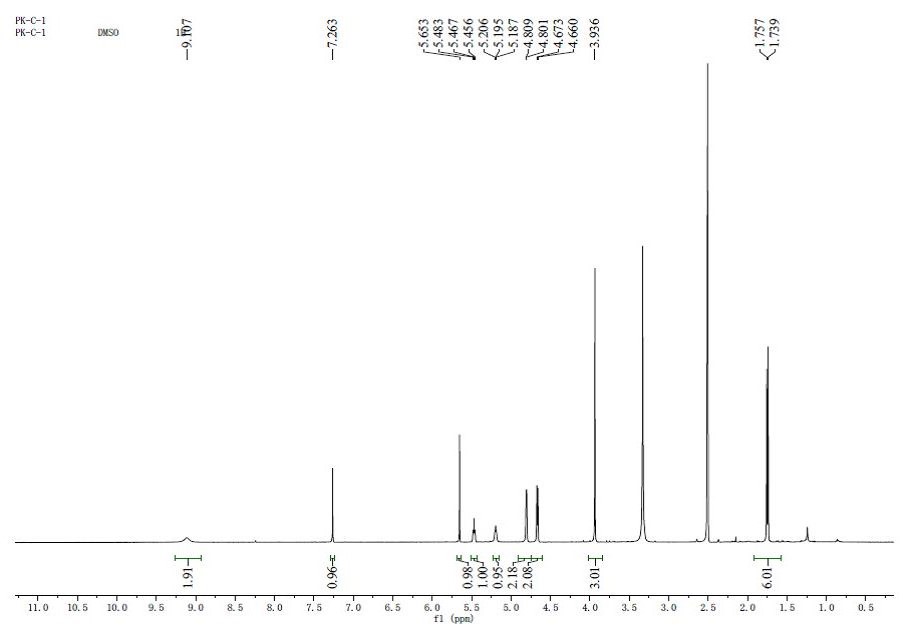


**Figure S16.** ^13^C (125 MHz) NMR spectrum of compound **7** in DMSO-d_6_


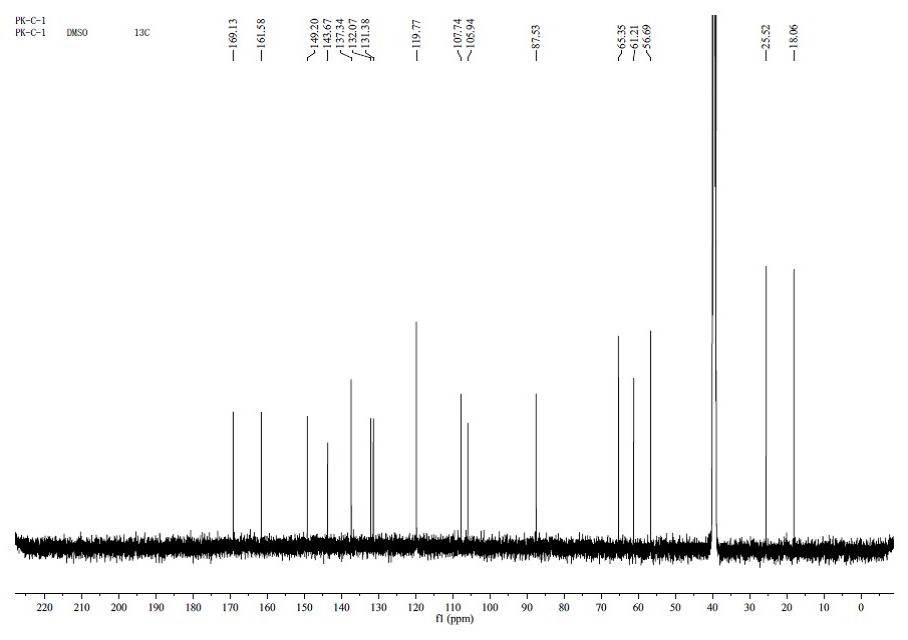


**Figure S17.** HSQC spectrum of compound **7** in DMSO-d_6_

**Figure S18.** HMBC spectrum of compound **7** in DMSO-d_6_

**Figure S19.** NOESY spectrum of compound **7** in DMSO-d_6_
